# Systematic analysis of glutamine metabolism family genes and exploration of the biological role of GPT in colorectal cancer

**DOI:** 10.18632/aging.205079

**Published:** 2023-10-17

**Authors:** Weiqi Dai, Wenhui Mo, Wenqiang Xu, Dengyu Han, Xuanfu Xu

**Affiliations:** 1 Department of Gastroenterology, Shidong Hospital Affiliated to University of Shanghai for Science and Technology, Shanghai, China

**Keywords:** colorectal cancer, machine learning, GPT, scRNA-seq, tumor microenvironment

## Abstract

Background: Colorectal cancer (CRC) is a malignancy of the digestive system with high incidence rate and mortality, and reliable diagnostic and prognostic markers for CRC are still lacking. Glutamine metabolism is crucial to the occurrence and development of CRC. However, no research has systematically analyzed the biological role of glutamine metabolism-related genes (GMRGs) in CRC.

Methods: We downloaded gene expression data and clinical data of CRC patients from the TCGA database. The UCSC database downloads pan-cancer gene expression data and prognosis data. Characteristic GMRGs were screened out using differential analysis and two types of machine learning (SVM-REF and RandomForest). Single-cell RNA-sequencing data from CRC patients were downloaded from GEO data. ROC curve was used to evaluate the diagnostic value. Kaplan-Meier method and univariate Cox regression analysis were used to evaluate the prognostic value. The oncopredict package is used to calculate IC50 values for common drugs in CRC patients.

Results: A total of 31 differentially expressed GMRGs were identified, 9 of which were identified as characteristic GMRGs. Further evaluation of diagnostic and prognostic value finally identified GPT as the most important GMRGs in CRC. scRNA-seq analysis revealed that GPT is almost exclusively expressed in epithelial cells. GPT expression is closely related to the tumor microenvironment and can effectively distinguish the sensitivity of different CRC patients to clinical drugs. In addition, pan-cancer analysis showed that GPT is an excellent diagnostic and prognostic marker for multiple cancers.

Conclusions: GPT is a reliable diagnostic, prognostic marker and therapeutic target in CRC.

## INTRODUCTION

As we all know, colorectal cancer (CRC) has a high incidence rate and mortality. According to statistics, in 2020, there will be about 1, 880, 000 new CRC cases and nearly 920, 000 CRC deaths worldwide [1]. Although great efforts have been made in early screening, which can effectively reduce the incidence of CRC [2], the morbidity and mortality of CRC have not been significantly improved, and the prognosis of patients with advanced CRC is still very poor [3]. It is estimated that there will be about 3, 200, 000 new CRC cases and 1, 600, 000 CRC deaths by 2040 [4]. At present, the mechanism of occurrence and development of CRC is still unclear. Therefore, it is particularly important to find new diagnostic and prognostic biomarkers for CRC patients. Metabolic reprogramming is generally considered to be one of the hallmarks of cancer [5].

In addition to classic glycolysis (Warburg effect) [6], researchers have focused on glutamine metabolism in recent years [7]. Studies have shown that glutamine is a non-essential amino acid with the highest content in blood [8]. In addition to providing a source of nitrogen, glutamine is a major source of carbon that supports cancer cell growth, especially when mitochondria are dysfunctional [9, 10]. Therefore, glutamine is one of the essential metabolites for cancer cells to maintain a malignant phenotype. Previous research has implicated that glutamine metabolism is associated with proliferation, invasion, autophagy, and immune evasion of cancer cells [11–15], and is a promising new target for cancer therapy [16, 17]. Therefore, a comprehensive analysis of GMRGs is imperative to find new promising biomarkers for the diagnosis and prognosis of CRC.

Glutamic pyruvic transaminase (GPT), also known as glutamic pyruvic transaminase 1, can reversibly catalyse alanine and α-ketoglutarate into pyruvate and glutamic acid and plays a pivotal role in the intermediate metabolism of amino acids and glucose [18]. Unlike mitochondrial GPT2, which has been extensively studied in cancer [19–21], GPT1 has been rarely reported in cancer. Current studies have shown that inhibiting GPT1 expression can reduce ATP production, thereby attenuating the malignant phenotype of HCC, which is a new therapeutic target for HCC [22]. In addition, GPT1 can be negatively regulated by SIRT4 to affect cervical cancer cell apoptosis by affecting glutamine metabolism. However, GPT1 has not been studied in other cancers, including CRC. Therefore, it is essential for the systematic study of GPT in CRC.

This study screened characteristic genes in the glutamine metabolism related family through multiple machine learning algorithms, and jointly analyzed their diagnostic and prognostic value, ultimately determining GPT as the key GMRGs in colorectal cancer. Then, we utilized bioinformatics to analyze the biological role of GPT in CRC and explore the relationship between its expression and tumor microenvironment (TME) and drug sensitivity. Finally, we also explored the differential expression, diagnostic performance, and prognostic value of GPT in pan cancer.

## MATERIALS AND METHODS

### Data download and processing

The RNA-seq expression data and survival information from the CRC cohort (TCGA-COAD/READ) were obtained from the TCGA database (https://portal.gdc.cancer.gov/). Expression data and prognostic information of 31 cancers for pancancer analysis were downloaded from UCSC database (https://xena.ucsc.edu/). Single-cell RNA sequencing (scRNA-seq) data of 12 CRC patients were downloaded from GSE166555. The scRNA-seq data in GSE166555 were downloaded from the Gene Expression Omnibus (GEO, https://www.ncbi.nlm.nih.gov/geo/) database. The glutamine metabolism-related genes involved in this study were obtained from previous studies published by He. et al. [23]. Analysis of the differential expression of GMRGs in the TCGA-CRC cohort using the R package “Limma”, and the screening condition was set to “|Foldchange > 2, P < 0.05|”.

### Machine learning screening for differentially expressed GMRGs

For the differentially expressed GMRGs, we further used SVM-REF and RandomForest algorithm for separation to screen out the characteristic genes. SVM-REF is a powerful classification algorithm [24], that can be applied to characterize cancer, allowing the identification of biomarkers for diagnosis. Similarly, RandomForest is also a powerful classification algorithm [25] that ranks gene importance, and in this study, we picked genes with an importance score greater than 2. Finally, we use the common genes identified by these two machine learning algorithms for further research and consider them as characteristic genes of CRC.

### scRNA-seq analysis of characteristic genes

The scRNA-seq data were preprocessed using the “Seurat” package. “PercentageFeatureSet” is used to calculate the percentage of mitochondrial genes. Cells with a gene count below 50 and a mitochondrial gene percentage above 5 were eliminated. The 1500 genes with the largest variation between cells were analyzed as cell clusters, the principal component was set to 20, and the resolution was 0.5.

### Evaluation of diagnostic performance of characteristic genes

We further used the receiver operating characteristic curve (ROC) based on specificity and sensitivity to evaluate the diagnostic performance of the characteristic gene. The AUC indicated the diagnostic performance of the characteristic gene. Different AUC values mean different diagnostic values [26]. AUC = 1.0 means perfect diagnostic value; AUC = 0.9-1.0 means high diagnostic value; AUC = 0.7-0.9 means relative diagnostic value; AUC = 0.5-0.7 means low diagnostic value; AUC = 0.5 means no diagnostic value.

### Evaluation of the prognostic value of characteristic genes

We performed a further analysis on the prognostic value of the characteristic genes. First, we observed the survival curves of different expression levels of the characteristic genes using the Kaplan-Meier method. Second, we further evaluated the ability of characteristic genes to affect the prognosis of CRC using univariate Cox regression analysis and displayed it using a forest plot. In the prognostic analysis, we excluded the samples with missing survival data and survival time ≤30.

### External validation and experimental validation of core gene GPT expression

The GEPIA2 database analyzed the differential expression of the key gene GPT at the mRNA level of colon cancer and rectal cancer online (http://gepia2.cancer-pku.cn/#index) [27]. The UCLCAN database analyzed the expression difference of GPT at the protein level in the CPTAC database online (http://ualcan.path.uab.edu/index.html) [28]. The HPA database (https://www.proteinatlas.org/) obtained the immunohistochemical images of GPT in normal colorectal and CRC tissues [29], and obtained the basic information of the corresponding tissue samples.

### CRC specimen collection, RNA extraction and quantitative real-time PCR (qRT-PCR) reaction

The 20 pairs of paracancerous normal tissues and matched CRC tissues were all from Shanghai Shidong Hospital. All patients provided written informed consent to participate in this study.

The qRT-PCR experiment was performed according to the previous procedure [30]. Briefly, total RNA from normal and CRC tissues was extracted using RNA-easy isolation reagent (Vazyme, China), and then the RNA was reversed to cDNA using PrimeScript™ RT Master Mix (Takara Bio, Japan). Finally, we performed qRT-PCR reactions using SYBR-Green qPCR Master Mix (Vazyme, China) and normalised using GAPDH. The experimental results were quantified relative to the 2-ΔΔCt method. Primer sequences were designed in this study as follows, The GAPDH primer sequences, forward: GTCTCCTCTGACTTCAACAGCG, reverse: ACCACCCTGTTGCTGTAGCCA. The GPT primer sequences, forward: CAAGAAGGTGCTCATGGAGATGG, reverse: TGTTCACCACCTCCACATAGCC.

### Co-expression analysis and enrichment analysis

The co-expression network of GPT was analyzed using the LinkedOmics database (http://www.linkedomics.org/). GO analysis and KEGG analysis explored the biological processes involved in the co-expression network.

### Assessment of the level of immune cell infiltration

Based on the gene expression profile of TCGA-CRC, we used CIBERSORT algorithm to evaluate the proportion of twenty-two types of immune cell [31], and samples with p < 0.05 were used for further analysis. Then, this research evaluated differences in the infiltration levels of 22 types of immune cells in CRC patients with different GPT expression.

### Somatic mutation spectrum analysis

We used the R package “maftools” [32] to analyze the somatic gene mutation profile of TCGA-CRC patient samples. First, we analyzed the mutation status of characteristic genes in TCGA-CRC samples. Second, we further analyzed the 10 most mutated genes in samples with different levels of GPT expression.

### Drug sensitivity analysis

We used R package “oncoPredict” [33] to analyze the half maximal inhibitory concentration (IC50) of TCGA-CRC samples based on gene expression data. The lower the IC50 value, the higher the drug sensitivity, and the larger the IC50 value, the lower the drug sensitivity.

### Pancancer analysis of GPT

As previously described, we used the R package “Limma” to explore the expression differences of GPT in pancancer. Likewise, the diagnostic performance of GPT in pancancer was evaluated using ROC curves. The prognostic value of GPT in pancancer was evaluated using univariate Cox analysis and Kaplan-Meier method.

### Statistical analysis

All bioinformatics research in this study was done by R language (version 4.1.2). Differences between groups were analyzed using the Wilcoxon test. The Kaplan-Meier method was used to draw survival curves. qRTPCR results were completed using Graphpad prism 9 software. p < 0.05 means that the results are statistically significantly different.

### Data availability statement

The original contributions presented in the study are included in the article/Supplementary Material, further inquiries can be directed to the corresponding authors.

## RESULTS

### Characteristic gene screening of GMRGs

**Figure 1:**
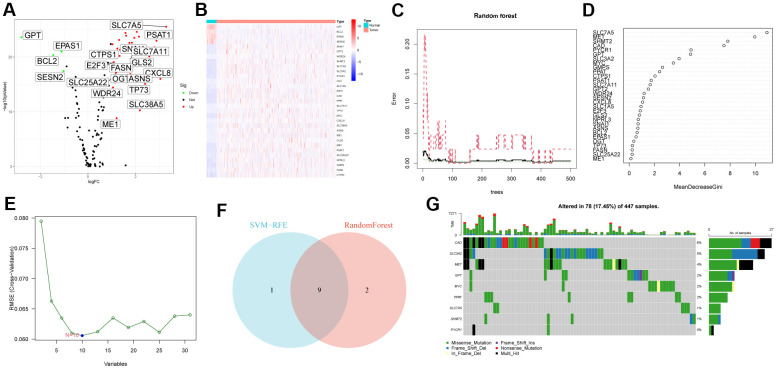
**Characteristic gene screening.** (**A**) Volcano map of differential expression of GMRGs. (**B**) Heatmap of DEG. (**C**, **D**) RandomForest evaluates the relative importance of DEG. (**E**) SVM–RFE algorithm. (**F**) Venn diagram of two machine learning algorithms. (**G**) Somatic mutation profiles of characteristic genes.

First, we analyzed the differential expression of 118 glutamine metabolism family genes in the TCGA-CRC cohort, and the results showed that 31 GMRGs were differentially expressed genes (DEG), of which 4 GMRGs were down-regulated and 27 GMRGs were up-regulated. Figure 1A shows the volcano map of GMRG as well as the heat map of DEG (Figure 1B). Then, we performed two machine learning algorithms on DEG to screen out characteristic gene: RandomForest (Figure 1C, 1D) and SVM-RFE (Figure 1E). Detailed results are detailed in Supplementary Table 1. According to the results of the above two machine learning algorithms, we used the Venn diagram to obtain 9 overlapping genes and defined them as characteristic gene (Figure 1F). Finally, we explored the mutation status of CRC signature genes and found that CAD (6%), SLC3A2 (5%) and MET (4%) had the highest mutation rates (Figure 1G).

### scRNA-seq analysis of characteristic genes

**Figure 2:**
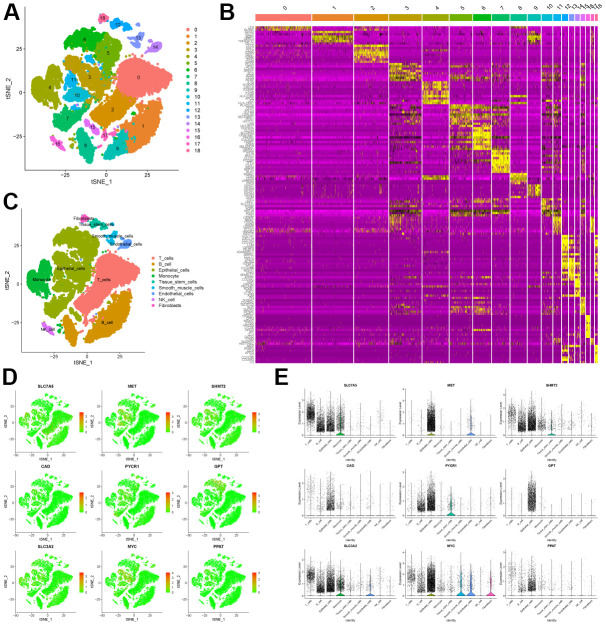
**scRNA-seq analysis of CRC tissue.** (**A**) tSNE analysis of CRC tissue scRNA-seq data to classify cell clusters. (**B**) Heatmap showing the top 10 genes highly expressed in each cell cluster. (**C**) The “Sin-gleR” package annotates the cell clusters into 9 cell types. (**D**) The distribution of the characteristic genes in the 9 cell types. (**E**) The bubble plot shows the expression levels of the characteristic genes in the 9 cell types.

First, we processed single-cell sequencing data of CRC cancer tissues to classify cells into 18 clusters (Figure 2A). We then displayed the 10 genes that were significantly overexpressed in each cluster in Figure 2B. In addition, we automatically annotated cell types through the “SingleR” package, and finally determined that there are 9 main types of cells in CRC tissues, including T cells, B cells, Epithelial cells, Monocyte, Tissue stem cells, Smooth muscle cells, Endothelial cells, NK cells, Fibroblasts (Figure 2C).

**Figure 3:**
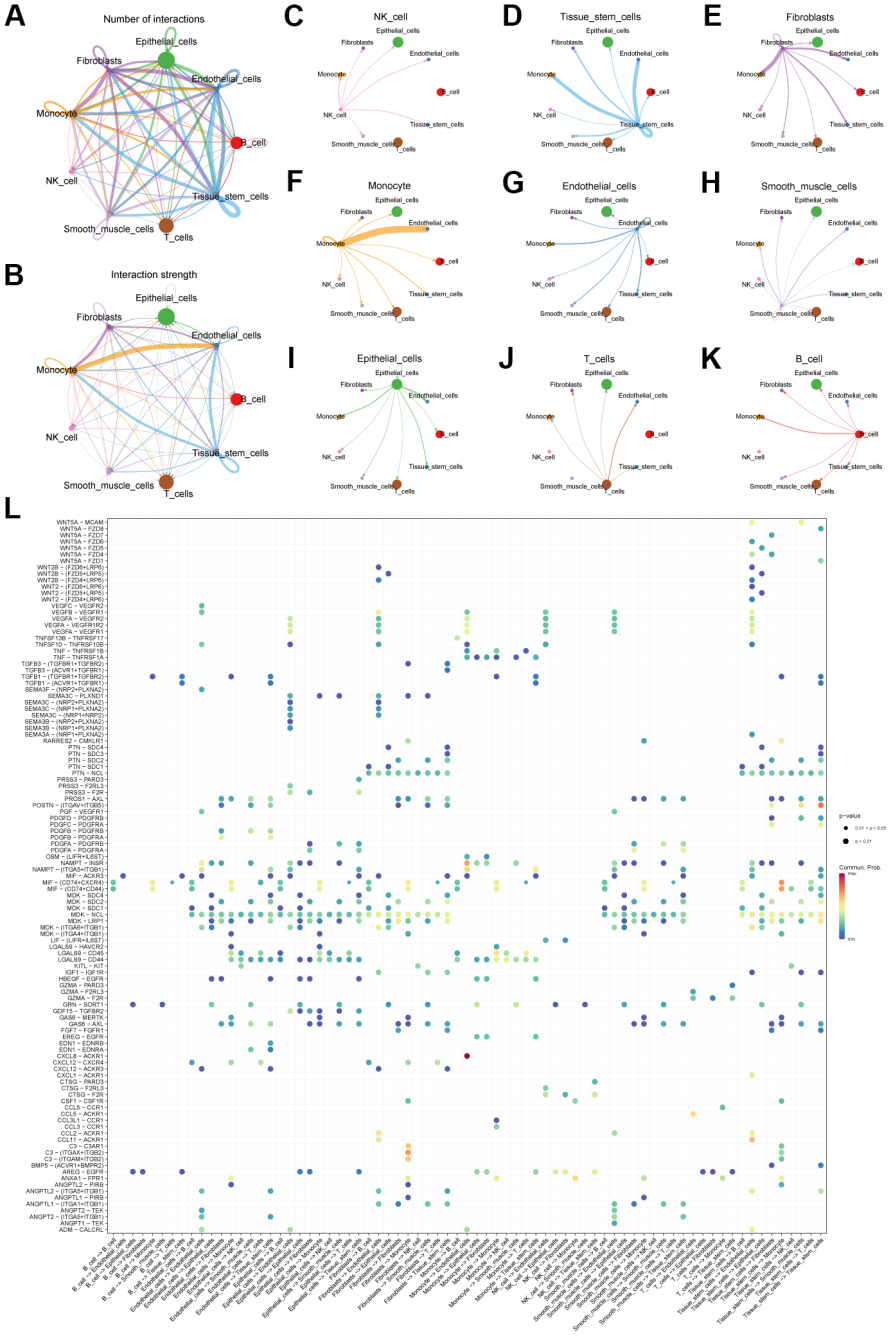
**Cell communication analysis of scRNA-seq data.** (**A**) Network diagram showing the number of connections between different cell types. (**B**) Network diagram showing the weight of connections between different cell types. (**C**–**K**) Diagram of the communication network between different cells and other cells. (**L**) Bubble plot showing genes involved in cell communication.

Second, we explored the connections between cells using cell communication analysis. We visualized the number (Figure 3A) and weight (Figure 3B) of interactions between the 9 cell types. And centering on each cell type, the communication relationship between it and 9 cell types is shown separately (Figure 3C–3K). Furthermore, we use heatmaps to visualize the ligand-receptor molecules that mediate interactions between cells (Figure 3L).

Finally, we explored the distribution of characteristic genes in the TME (Figure 2D). Bubble plots show the expression levels of signature genes in each cell type (Figure 2E). Combining the above results, we found that SLC7A5 was highly expressed in monocyte, B cells and T cells. MET is mainly expressed in endothelial cells and epithelial cells. SHMT2 has similar expression level in other cell types except T cells. CAD and PPAT were expressed at low levels in all cell types. PYCR1 is highly expressed in tissue stem cells, epithelial cells and fibroblasts. GPT is almost exclusively expressed in epithelial cells. SLC3A2 was highly expressed in monocytes, but very low in fibroblasts. MYC is mainly expressed in endothelial cells, smooth muscle cells and epithelial cells.

### Diagnostic and prognostic value of characteristic genes

First, we analyzed the diagnostic performance of the characteristic genes using ROC curves. We found that the 9 characteristic genes all had high AUC values (Figure 2A–2I), specifically: CAD (AUC = 0.976), GPT (AUC = 0.973), MET (AUC = 0.983), MYC (AUC = 0.974), PPAT (AUC = 0.962), PYCR (AUC = 0.972), SHMT2 (AUC = 0.981), SLC3A2 (AUC = 0.968) and SLC7A5 (AUC = 0.992). These results imply that all characteristic genes are a potentially powerful class of diagnostic markers.

At the same time, we analyzed the survival curves of the 9 characteristic genes with different expression levels using the Kaplan-Meier method (Figure 2J–2U). We found that high expression of CAD and SLC7A5 had a worse prognosis, while high expression of GPT, MET, MYC, and PPAT had a better prognosis. The results showed that high expression of CAD and SLC7A5 had worse prognosis, while high expression of GPT, MET, MYC and PPAT had better prognosis. However, the prognosis of patients with different expression levels of PYCR, SHMT2, and SLC3A2 was not significantly different.

**Figure 4:**
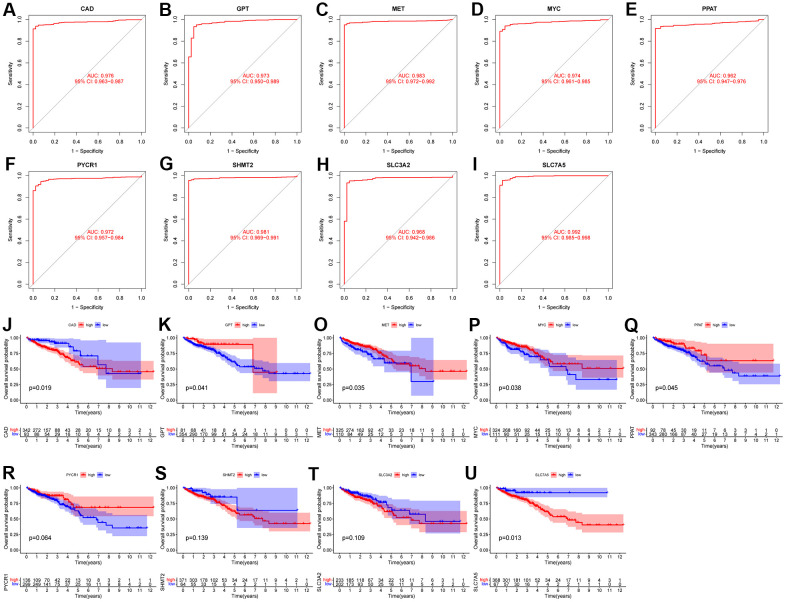
**Evaluation of diagnostic performance and prognostic value of characteristic genes.** (**A**–**I**) ROC curves of 9 characteristic genes. (**J**–**U**) KM survival curves of 9 characteristic genes.

Finally, we used univariate Cox analysis and found only GPT as a protective factor affecting the prognosis, and none of the remaining 8 characteristic genes were factors affecting the prognosis in CRC (Figure 4A). Combining the above results, we regard GPT as the most important GMRGs in CRC and conduct further analysis.

### Co-expression network and enrichment analysis of GPT in CRC

**Figure 5:**
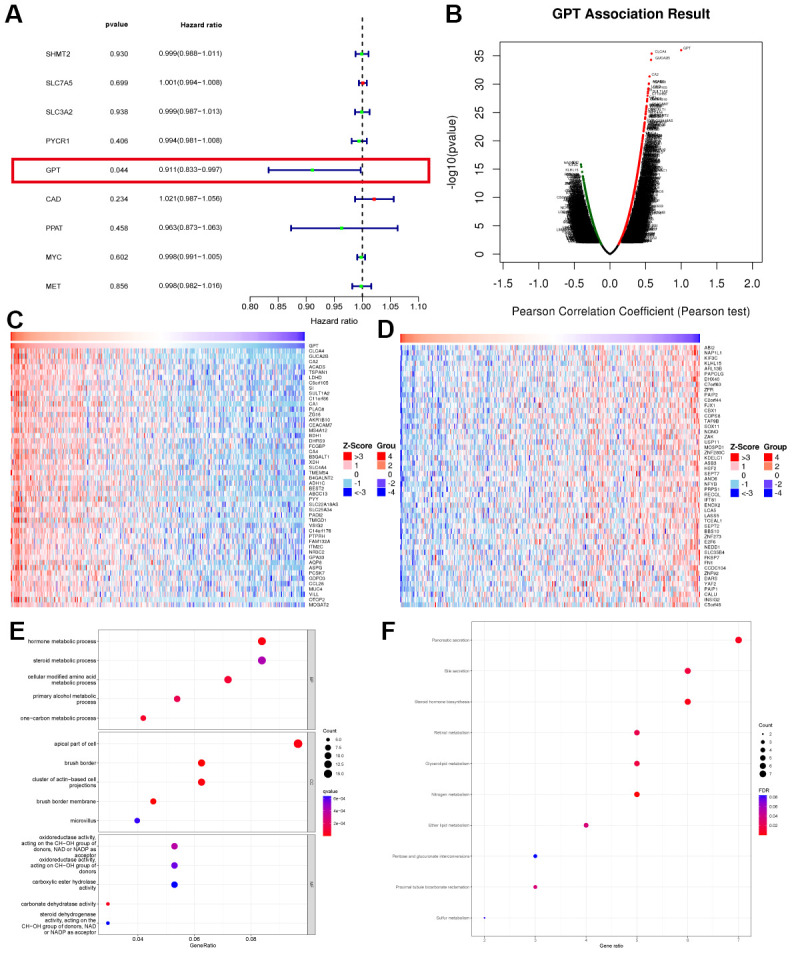
**Co-expression analysis and enrichment analysis.** (**A**) Univariate Cox regression analysis of characteristic genes. (**B**) Correlation of GPT ex pression with other genes. (**C**) Heatmap showing the 50 genes with the strongest positive correlation with GPT expression. (**D**) Heatmap showing the 50 genes most negatively correlated with GPT expression. (**E**) GO analysis. (**F**) KEGG analysis.

In order to fully understand the biological function of GPT in CRC, we analyzed the co-expression network of GPT in the LinkedOmics database online. The results showed that GPT expression was positively correlated with 3462 genes and negatively correlated with 5207 genes (Figure 5B). The heatmap shows the top 50 most significantly positively and negatively correlated genes with GPT expression, respectively (Figure 5C, 5D). We further performed GO analysis and KEGG analysis on the top 200 significantly positively correlated genes. The bubble charts show the top 15 GO analysis terms (including 5 BP, CC and MF terms respectively) and the 10 KEGG analysis terms (Figure 5E, 5F). GO analysis results showed that, in terms of BP, mainly enriched in hormone metabolic process; one−carbon metabolic process; cellular modified amino acid metabolic process; primary alcohol metabolic process; steroid metabolic process. in terms of CC, mainly enriched in brush border; brush border membrane; cluster of actin−based cell projections; apical part of cell; microvillus. in terms of MF, mainly enriched in carbonate dehydratase activity; oxidoreductase activity, acting on the CH−OH group of donors, NAD or NADP as acceptor; oxidoreductase activity, acting on CH−OH group of donors; steroid dehydrogenase activity, acting on the CH−OH group of donors, NAD or NADP as acceptor; carboxylic ester hydrolase activity. KEGG analysis mainly enriched in Pancreatic secretion; Bile secretion; Steroid hormone biosynthesis; Retinol metabolism; Glycerolipid metabolism. In short, the results suggest that GPT co-expressed genes are mainly involved in various metabolic pathways.

### External validation and experimental validation of GPT expression

**Figure 6:**
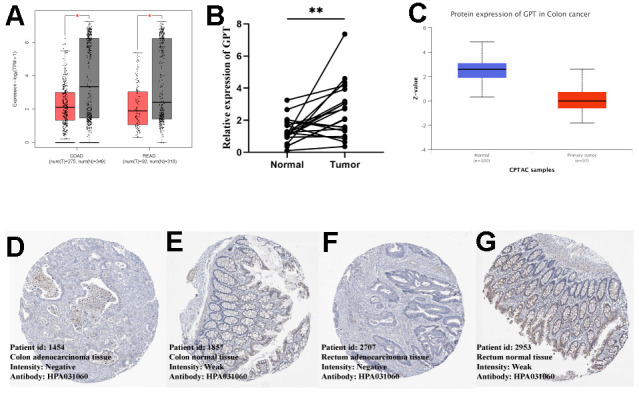
**External data and experimental data verify GPT expression of GPT in CRC.** (**A**) GEPIA2 database showing differential expression of GPT in COAD and READ (mRNA level). (**B**) qRT-PCR showed that GPT was significantly lower in CRC tissues than in adjacent normal tissues. (**C**) The UALCAN database showed that the low expression of GPT in COAD was correlated with cancer tissues (protein level). (**D**, **E**) HPA database showing IHC images of GPT in COAD and colon normal tissues. (**F**, **G**) HPA database showing IHC images of GPT in READ and rectal normal tissues.

We first explored the differential expression of GPT in COAD and READ using the GEPIA2 database. We found that GPT mRNA expression levels were low in COAD and READ (Figure 6A). In addition, we detected GPT mRNA levels of GPT using qRT-PCR. The results also showed that compared with CRC tissues, GPT was highly expressed in normal tissues (Figure 6B). In addition, we also evaluated the expression difference of GPT at the protein level. First, we used the CAPTC database on UALCAN to analyze the differences in GPT protein expression levels, and found that GPT protein levels were significantly decreased in COAD tissues compared with normal tissues (Figure 6C). In addition, we obtained immunohistochemical images of normal colon and rectal tissues and CRC tissues from the HPA database. We found that the intensity of GPT in CRC tissues was significantly reduced compared to normal tissues (Figure 6D–6G).

### Association analysis between GPT expression and TME

**Figure 7:**
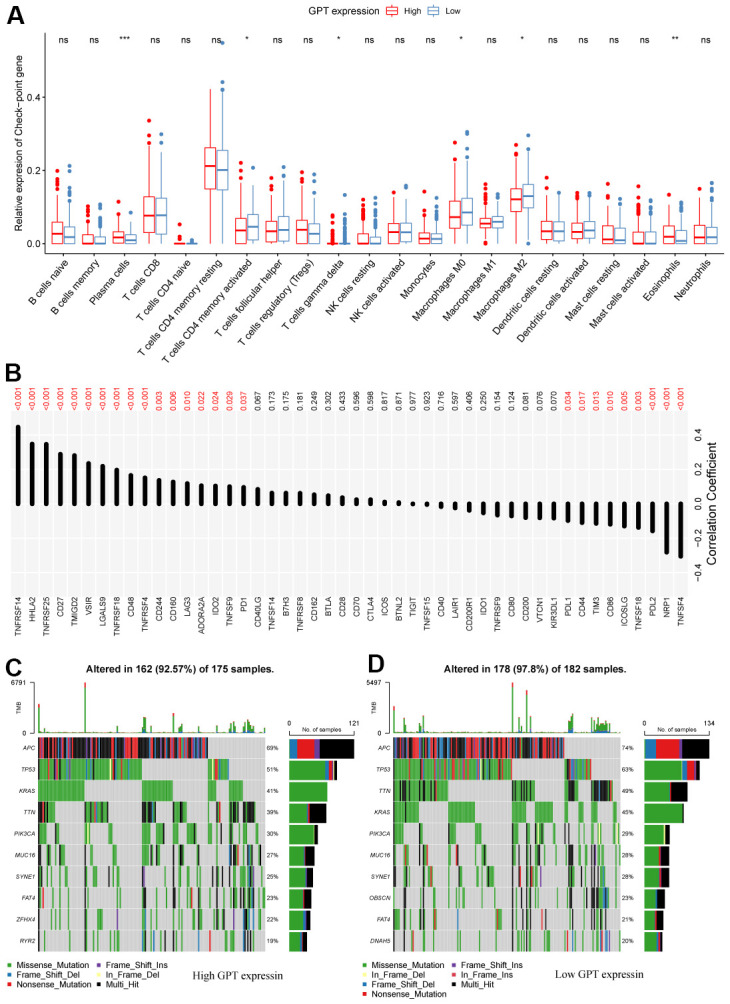
**Correlation between GPT expression and the tumor microenvironment.** (**A**) Differential analysis of immune cell infiltration levels with different levels of GPT expression. (**B**) Correlation between GPT expression and immune checkpoint molecules. (**C**, **D**) Somatic mutation profiles at different GPT expression levels.

Our research assessed the infiltration levels of 22 immune cells in TCGA-CRC tissue samples using the CIBERSORT algorithm. We found that compared to the GPT low expression group, the infiltration levels increased significantly increased in the GPT high expression group, while the infiltration levels of T cells CD4, gamma delta of T cells gamma delta, Macrophages M0 and Macrophages M2 decreased significantly decreased (Figure 7A).

We also analyzed the correlation between GPT expression and 48 immune checkpoint molecules (ICM), in which GPT expression was correlated with 26 ICM, including positive correlations with 17 ICM and negative correlations with 9 ICM (Figure 7B).

Furthermore, we explored the somatic mutation profile in patients with CRC and identified the 10 most mutated genes in different GPT expression level groups. The results showed that APC, TP53, KRAS, TTN, PIK3CA, MUC16, and SYNE1 were the 8 most mutated genes in different groups of expression level groups, and APC had the highest mutation rate (Figure 7C, 7D).

### Prediction of potentially sensitive drugs

**Figure 8:**
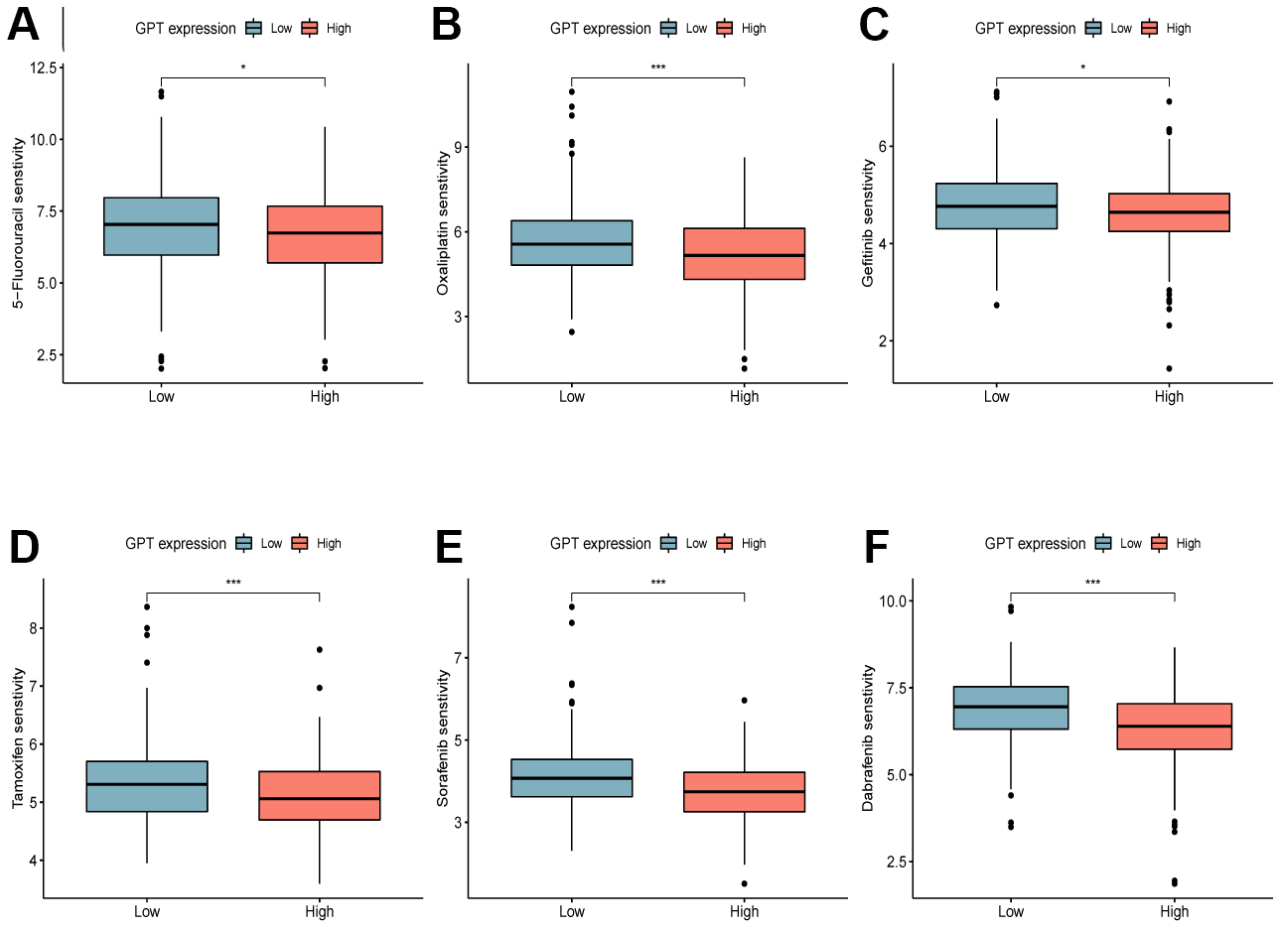
**Drug sensitivity analysis.** Sensitivity analysis of 5-Fluorouracil (**A**), Oxaliplatin (**B**), Gefitinib (**C**), Tamoxifen (**D**), Sorafenib (**E**), Dabrafenib (**F**) in different GPT expression levels.

In order to explore the sensitivity of patients with different GPT expression groups to common chemotherapy drugs, we used the R package “oncoPredict” to analyze the IC50 values of CRC patients in the TCGA-CRC cohort to 5−Fluorouracil, Oxaliplatin, Gefitinib, Tamoxifen, Sorafenib and Dabrafenib. The results showed that the IC50 values of 5−Fluorouracil (Figure 8A), Oxaliplatin (Figure 8B), Gefitinib (Figure 8C), Tamoxifen (Figure 8D) and Sorafenib (Figure 8E) were higher in the GPT low expression group, whereas those of Dabrafenib (Figure 8F) were lower.

### Diagnostic performance of GPT in pancancer

**Figure 9:**
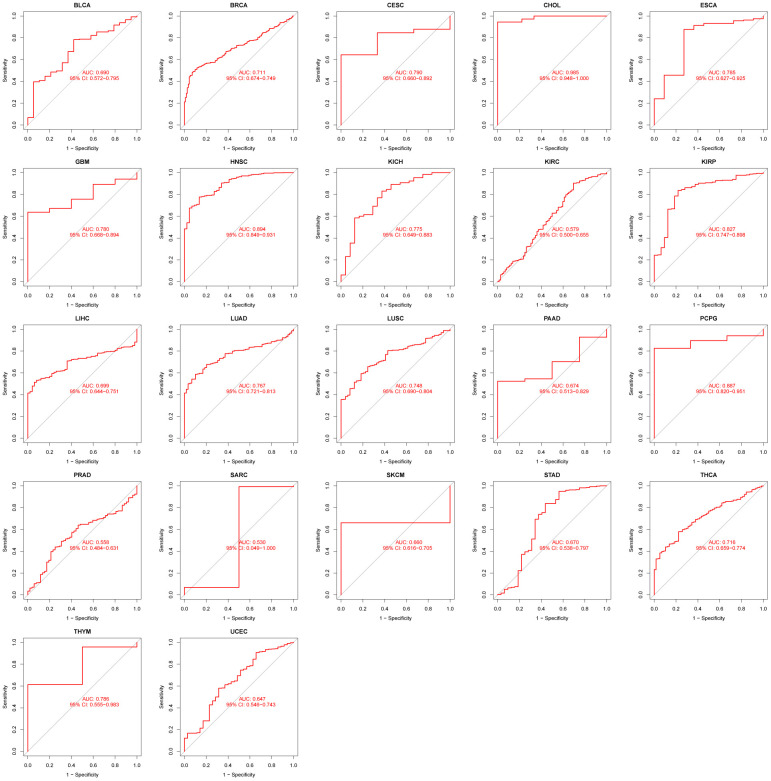
RUC curve to evaluate the diagnostic performance of GPT expression in 22 cancer types.

As mentioned above, the diagnostic performance of GPT in pancancer is evaluated by the ROC curve. We found that GPT had excellent diagnostic performance in CHOL (AUC: 0.985). In BRCA (AUC: 0.711), CESC (AUC: 0.790), ESCA (AUC: 0.785), GBM (AUC: 0.780), HNSC (AUC: 0.894), KICH (AUC: 0.775), KIRP (AUC: 0.827), LUAD (AUC: 0.767), LUSC (AUC: 0.748), PCPG (AUC: 0.887), THCA (AUC: 0.716) and THYM (AUC: 0.786) had higher diagnostic performance (Figure 9).

### Expression of GPT in pancancer

**Figure 10:**
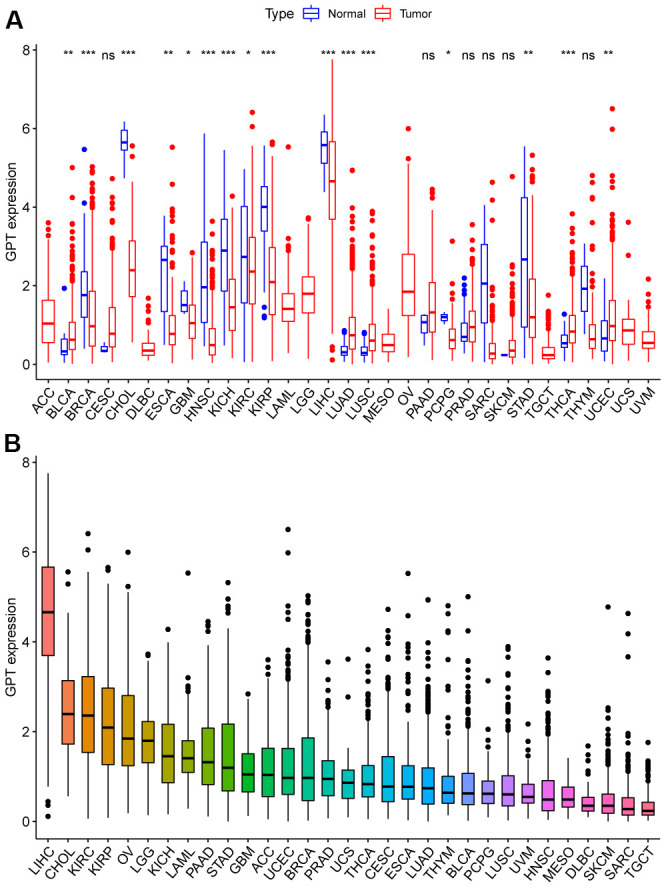
**GPT expression patterns in pancancer.** (**A**) Different expression of GPT in pancancer. (**B**) Ranking map of GPT expression in tumor tissues (from high to low).

GPT was differentially expressed in 16 cancers compared to normal tissues. Specifically, GPT was low expressed in BRCA, CHOL, ESCA, GBM, HNSC, KICH, KIRC, KIRP, LIHC, LUAD, LUSC, PCPG, STAD, UCS, and highly expressed in BLCA, LUAD, LUSC, THCA (Figure 10A). Furthermore, we sorted the expression of the tumor tissues, we found that GPT had the lowest expression level in TGCT and the highest expression level in LIHC (Figure 10B).

### Prognostic value of GPT in pancancer

**Figure 11:**
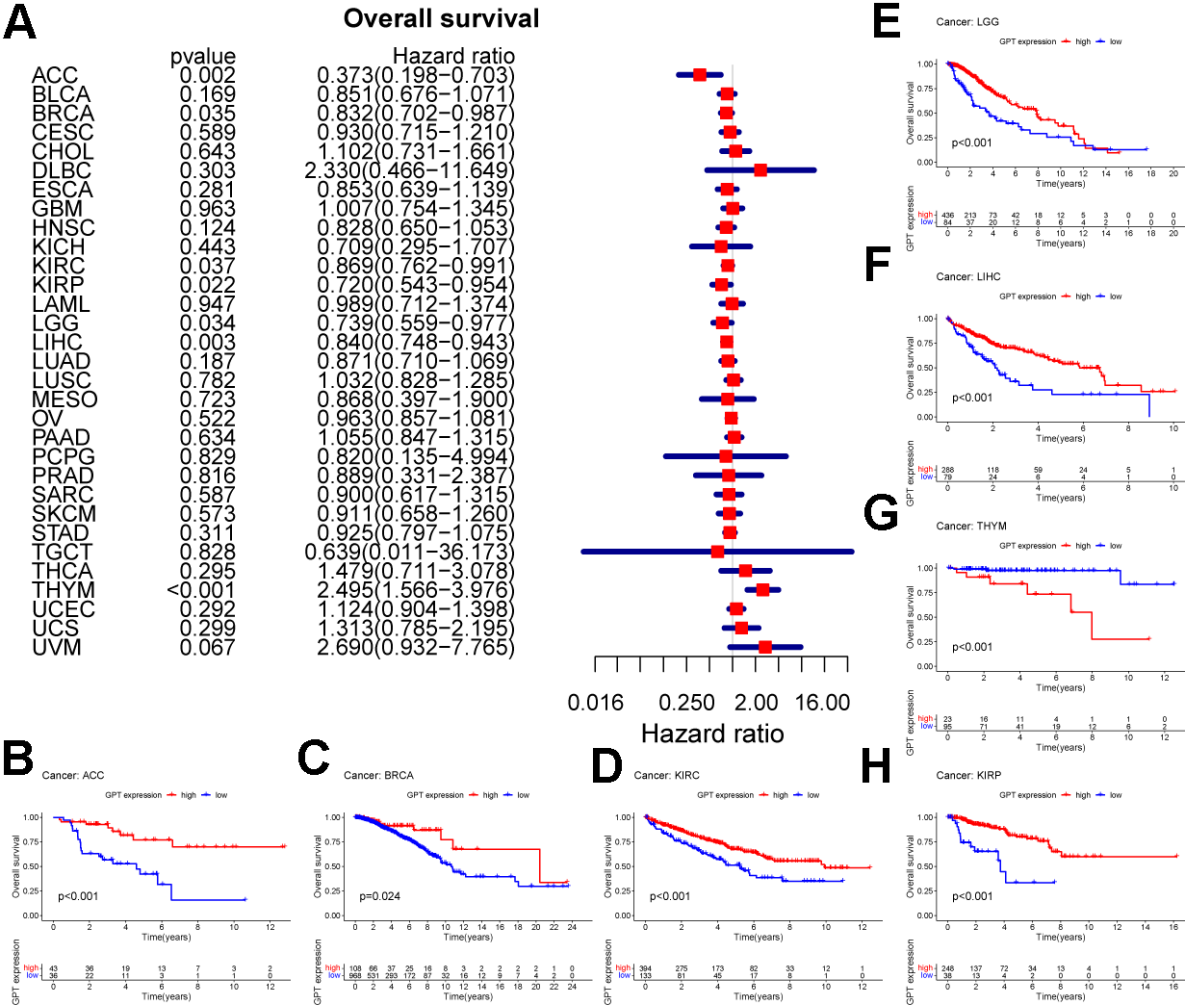
**Prognostic value of GPT in pancancer.** (**A**) Univariate Cox analysis to evaluate the correlation between GPT expression and OS in the patient. (**B**–**H**) Survival curves of GPT expression in 7 cancers.

As previously mentioned, we explored the role of GPT expression in the prognosis of pancancer using univariate Cox analysis and drew a forest plot. We found that GPT expression can affect the prognosis of ACC, BRCA, KIRC, KIRP, LGG, LIHC, and THYM. Specifically, GPT acts as a protective factor affecting the prognosis of ACC (HR: 0.373), BRCA (HR: 0.832), KIRC (HR: 0.869), KIRP (HR: 0.720), LGG (HR: 0.739), LIHC (HR: 0.840) and acts as a risk factor affecting the prognosis of THYM (HR: 2.495) (Figure 11A). Finally, for these 7 cancer types, we used the Kaplan-Meier method to analyze the survival curves of different expression levels of GPT, and the results showed that the GPT high expression group had a better prognosis in ACC (Figure 11B), BRCA (Figure 11C), KIRC (Figure 11D), KIRP (Figure 11E), LGG (Figure 11F), LIHC (Figure 11G), and in THYM (Figure 11H) The prognosis is poor.

## DISCUSSION

CRC is a malignant tumour of the digestive system with high incidence rate, and its morbidity and mortality are still on the rise. CRC is a highly heterogeneous malignant tumor, so it is necessary to find new biomarkers as the diagnosis, prognosis, and treatment target of CRC. Current research shows that glutamine metabolism plays a key role in the growth, metastasis, recurrence and drug resistance of CRC [34–36]. Therefore, in this study, we systematically analyzed the GMRGs in order to find powerful and promising biomarkers for diagnosis and prognosis. In this study, we first performed differential analysis on 113 GMRGs, and found that a total of 27 GMRGs were highly expressed in the TCGA-CRC cohort, and 4 GMRGs were lowly expressed. Then, we performed two machine learning algorithms, SVM-REF and RandomForest, on the 31 DEG to find more representative eigengenes. Based on the two algorithms, we found that 9 GMRGs can be regarded as characteristic genes of CRC. Furthermore, we further explored the cellular composition of the tumor microenvironment in CRC in order to understand the heterogeneity of CRC tissue. The results showed that CRC tissue was mainly composed of 9 types of cells, and epithelial cells, B cells, and T cells dominated, suggesting that immune cells may play an important role in the occurrence and development of CRC. We also explored the expression of 9 GMRGs in cells. Notably, GPT is expressed almost exclusively in epithelial cells. On this basis, we explored the prognostic value of signature genes by Kaplan-Meier method and univariate Cox analysis, and found that only GPT showed some prognostic value in the TCGA-CRC cohort. CRC patients with high GPT expression had a better prognosis than those with low GPT expression and were a protective factor for the prognosis of CRC. Based on this, we evaluated the expression of GPT in CRC again, and we double verified the low expression of GPT in CRC at the mRNA level and protein level by GEPIA2, UALCAN, HPA and qRT-PCR experiments. In addition, GO analysis and KEGG results based on GPT co-expressed genes indicated that GPT may affect multiple metabolic pathways.

Previous studies have shown that glutamine metabolism and the tumor immune microenvironment closely interact with each other [15, 37]. Interestingly, we also found that GPT expression was associated with the tumor microenvironment in CRC through bioinformatics. First, in our study, we found that GPT expression can affect the infiltration levels of various immune cells through the CIBERSORT algorithm. Studies have shown that Plasma cells and Eosinophils play an important role in anti-tumor, which means better clinical outcomes [38–41]. Whereas Macrophages M2 generally have tumor-promoting effects [42–44], implying a worse prognosis. These results may explain the better prognosis of low GPT expression in CRC. Immunotherapy is currently the mainstay of cancer treatment, and drug development based on immune checkpoint inhibitors is a promising strategy. Therefore, our research found that GPT expression was negatively correlated with 9 ICM and positively correlated with 17 ICM. This close correlation suggests that GPT may also be a promising immunotherapy marker. Finally, we explored the somatic mutations in different GPT expression levels, and found that the mutation rates of TP53 and TTN were decreased in the GPT high expression group compared with the GPT low expression group (TP53: 51% vs. 63%, TTN: 39% vs. 49%). Previous studies have shown that TP53, TTN mutations usually mean poorer prognosis in CRC [45, 46]. This may also be one of the high-expression group of reasons why the GPT had a better prognosis than the low-expression group of GPT.

We also predicted the correlation between GPT expression and the efficacy of common drugs. In this study, we predicted the sensitivity of 5-FU and Oxaliplatin, which are widely used in the first-line treatment of CRC [47, 48]. However, due to the frequent drug resistance events, we also analyzed the sensitivity of other common chemotherapeutic drugs. These results may provide potential therapeutic drugs for CRC patients with different levels of GPT expression.

Given that GPT is rarely reported in cancer, we also performed a pancancer analysis of GPT. First, we found that GPT was significantly differentially expressed in a variety of cancers (16/33). On the whole, GPT expression was reduced in most cancers. Through the ROC curve, we found that GPT showed relatively excellent diagnostic performance in 13 cancer types. Taken together, these results suggest that GPT may show promising promise in pancancer diagnostics. Finally, we also investigated the correlation between GPT expression and pancancer prognosis and found that GPT expression was a protective factor in 6 types of cancer and a risk factor in THYM. It shows that GPT plays different functions in different types of cancer.

This study, like other studies, has some limitations. First of all, this study did not verify the carcinogenesis of GPT in CRC through *in vivo* and *in vitro* experiments. Second, the specific mechanism by which GPT affects CRC has not been elucidated. Our future research will focus on the carcinogenesis and mechanism of GPT in CRC.

In summary, our systematic analysis of GMRGs screened for GPT as a potential diagnostic and prognostic marker for CRC, revealed the correlation between GPT and the tumor microenvironment, and explored the relationship between GPT expression and sensitivity to common drugs. These correlations between them suggest that GPT may be a potential therapeutic target for CRC. Finally, we also explored the diagnostic performance and prognostic value of GPT in pancancer, providing new insights for the function of GPT in pancancer.

## Supplementary Material

Supplementary Table 1
