# Supplementary material for: Systematic analysis of glutamine metabolism family genes and exploration of the biological role of GPT in colorectal cancer
Source: Aging (Albany NY). 2023 Oct 17;15(21):11811–30. doi: 10.18632/aging.205079 (PMC10683594; doi:10.18632/aging.205079)
Supplement: Supplementary Table 1 [file aging-15-205079-s001.pdf]

SUPPLEMENTARY TABLE

Supplementary Table 1. Detailed results of RandomForest and SVM-RFE.

SVM-RFE.gene	RandomForest.gene
SLC7A5	SLC7A5
MET	MET
SHMT2	SHMT2
SLC3A2	CAD
CAD	PYCR1
GPT	GPT
PYCR1	SLC3A2
MYC	MYC
PPAT	GMPS
PSAT1	PPAT
	CTPS1
